# Opening Up: Clients’ Inner Struggles in the Initial Phase of Therapy

**DOI:** 10.3389/fpsyg.2020.591146

**Published:** 2020-12-15

**Authors:** Gøril Solberg Kleiven, Aslak Hjeltnes, Marit Råbu, Christian Moltu

**Affiliations:** ^1^District General Hospital of Førde, Førde, Norway; ^2^Department of Clinical Psychology, University of Bergen, Bergen, Norway; ^3^Department of Psychology, University of Oslo, Oslo, Norway; ^4^Western Norway University of Applied Sciences, Bergen, Norway

**Keywords:** emotion in psychotherapy, interpersonal process recall, qualitative, holding back, process research

## Abstract

**Objective:**

To explore how clients in clinical settings experience the process of opening up and sharing their inner experiences in the initial phase of therapy.

**Methods:**

Two psychotherapy sessions of clients (*N* = 11) were videotaped and followed by interviews. Interpersonal process recall was used to obtain in-depth descriptions of clients’ immediate experiences in session. A follow-up interview was conducted 3 months later. The interviews were analyzed using thematic analysis.

**Results:**

The data revealed how and why clients distanced themselves from inner experiences in the initial phase of therapy. The overarching theme was “Holding back and struggling to open up,” which included four subthemes: (a) fearing the intensity and consequences of negative emotions; (b) experiences of being incapable and bodily stuck; (c) being insecure about one’s worthiness and right to share inner experiences with the therapist; and (d) struggling with feeling disloyal to loved ones.

**Conclusion:**

The participants held back because they feared different consequences of opening up. A range of concerns led participants to distance themselves from their inner experiences and/or to refrain from openly talking about them to the therapist. Concerns related to appropriate interpersonal conduct as client were especially important. This knowledge is highly relevant to clinicians when building safety for psychotherapeutic work.

## Introduction

The therapeutic process in psychotherapy is aimed at facilitating clients’ capacity to approach, recognize and reflect upon challenging life issues with openness and authenticity (Kolden et al., 2018). However, the process of *opening up* in treatment sessions is far from straightforward, as apprehension, shame or fright can prevail (e.g., MacFarlane et al., 2015; Baumann and Hill, 2016; Marks et al., 2019). It is natural that disclosure of personal material can take some time, sometimes opening up may occur only after months and sometimes it never happens (Farber, 2003).

In addition to opening up verbally to the therapist, various treatment orientations emphasize that it is necessary for the client to open up to and engage with personal issues internally, even when this is highly painful. Research shows that, across symptom categories and treatment orientations, emotional arousal coupled with cognitive-reflective exploration in treatment is positively correlated with treatment outcomes (Lane et al., 2014; Barbosa et al., 2017; Pascual-Leone and Yeryomenko, 2017). Different treatment orientations encourage the client to approach emotional experiences, within levels they are able to tolerate in the session, to further enhance the capacity to process emotions (Whelton, 2004; Peluso and Freund, 2018). Emotion oriented orientations have both commonalities and differences in what is considered particularly important when working with emotions in session. For example, Psychodynamic therapies recognize in-depth emotional exploration as essential (Diener et al., 2007; Subic-Wrana et al., 2016), contemporary third-wave Cognitive-behavioral therapies emphasize awareness and acceptance of emotions (Sloan and Kring, 2007), and Emotion-focused therapy (EFT) argues that clients need to get in touch with their core maladaptive emotional states to be able to transform them (Greenberg and Goldman, 2019).

Little scientific research has focused on how clients experience the process of opening up to emotion-ladened topics in psychotherapy. Clients’ *intrapersonal* processes have received little attention in research on clinically relevant mechanisms in psychotherapy (Pascual-Leone and Yeryomenko, 2017). Moreover, most process research has been conducted by external coders rating verbally expressed material (Swift et al., 2017). Rennie (1994a, c) and Levitt (2001, 2002) explored clients’ covert processes that could hinder or facilitate treatment progress, by the use of interpersonal process recall. Both have found that clients privately explore or distance themselves from emotional content in session. Hence, the client perspective seems valuable in research on clients’ experiences when opening up in therapy, as important processes not necessarily are shared with the therapist for a variety of reasons. This would be in line with the shift over the past two decades toward greater recognition of the client as an active participant in psychotherapy; the clients’ contributions and experiences in sessions have been shown to be increasingly important to the outcome of treatment in psychotherapy research (Bohart and Wade, 2013; McAleavey and Castonguay, 2015; Levitt et al., 2016). Moreover, understanding the active role of the client in psychotherapy also underscores how any client will approach the therapeutic encounter with a sensibility toward what is expected from them interpersonally in this setting, which might be unclear in initial phases.

Qualitative research that aim to explore clients’ general experiences in psychotherapy have largely been conducted post-session or post-treatment, by asking clients to recall events retrospectively. In these interviews, momentary experiences during the flow of events that occur during a session might be forgotten by the clients or might merge into more global impressions (Rodgers and Elliott, 2015). Emotion episodes in therapy might be passing events lasting only for seconds at a time, and clinically relevant information about important micro-processes might be lost in post-session recalls. A close examination of moment-to-moment changes in sessions is needed to gain more comprehensive clinical knowledge about clients’ experiences when opening up.

Moments where clients approach their inner experiences and explore these verbally and non-verbally with the therapist are shown to build and strengthen the therapeutic relationship (Timulak and Keogh, 2017; Gelso et al., 2018), and the therapeutic relationship is well documented as important to the outcome of therapy (Flückiger et al., 2018). Moreover, studies show that it might be beneficial when the therapeutic alliance is established early—e.g., by the fifth session of therapy (Bohart and Wade, 2013). Hence, clients’ experiences when encountering emotions in initial stages of therapy seem to be an important avenue for research. The aim of this study was to explore clients’ in-session experiences with important emotional moments in the initial phase of psychotherapy, to better understand critical incidents in therapy that may facilitate or hinder progress in treatment. The research question was: How do clients in ordinary clinical settings experience the process of opening up to and sharing their inner experiences in the initial phase of therapy?

## Materials and Methods

### Participants and Recruitment

#### Therapists

Treatment providers received information about the study at staff meetings or by e-mail and were encouraged to recruit clients. Participant therapists worked in institutions required to offer clinical treatment in line with national guidelines for best practice (American Psychological Association, Presidential Task Force on Evidence-Based Practice, 2006). No restriction was placed on theoretical orientation. The therapists who volunteered to recruit participants consisted of two specialized clinical psychologists, two clinical psychologists, and two clinical psychologist trainees, with 1–10 years of clinical practice. Five of the therapists worked at the (Department of Psychiatry, District General Hospital of Førde, Norway), and one therapist worked at a private institution (The Norwegian Institute of Emotion-Focused Therapy, Bergen). Four clinical treatment orientations were represented: Focused psychodynamic therapy (1 therapist); emotion-focused therapy (2 therapists); intensive short-term dynamic therapy (1 therapist); and integrative forms of therapy (2 therapists). The therapists where instructed to ask only new clients to consider participation, and exclude former clients. Sixteen clients were approached for participation over a period of 12 months; of these, five declined. Six treatment providers recruited 1–3 clients each.

#### Clients

Three male and eight female clients volunteered, ranging in age from 18 to 63 years. Eight clients were referred by first-line services to public outpatient services, to be eligible for therapy they needed to have a diagnosable disorder with moderate to severe functional impairment; we did not collect the diagnostic information from the formal assessment that established this eligibility. Three clients were self-referred to a private institute. The exclusion criteria for the study were acute psychosis, a primary diagnosis of addiction, known neuropsychological damage, or a global assessment of functioning and symptoms (GAF-F/GAF-S) below 45. We did not exclude participants who had previous experiences with therapy, due to the naturalistic design. About half of the participants had previous experiences. When asked in the interviews, clients described themselves as suffering from mild and moderate depression, anxiety, post-traumatic stress disorder, psychosomatic disorders, and borderline personality disorder. The clients and the therapist at the private institution were compensated for their participation; the clients were compensated for two sessions of therapy, and the therapist was compensated for 1 h of administrative work.

#### Researchers

The first author is a clinical psychologist and a research fellow with 3 years of clinical experience. The second author is an associate professor in clinical psychology with 10 years of clinical practice. The third author is an associate professor in clinical psychology with 23 years of clinical practice. The fourth author is a professor in clinical psychology with 13 years of clinical practice. Three of the four researchers have completed a 3-year training program in EFT, and all of them share an interest in process research and humanistic-psychodynamic, experiential-humanistic treatment approaches. None of the researchers had any treatment relationship with the clients in the study.

### Data Collection

**FIGURE 1:**
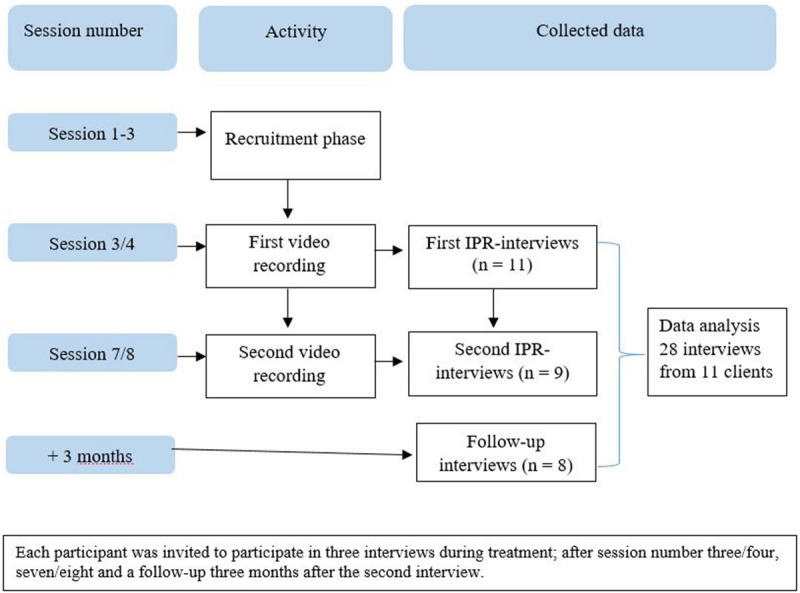
Data collection procedure. Each participant was invited to participate in three interviews during treatment, after session number three/four, seven/eight and a follow up 3 months after the second interview.

Clients’ descriptions of experiences in the initial phase of psychotherapy were gathered cross-sectionally by the use of interpersonal process recall (IPR) (Elliott, 1986). IPR was used to study microprocesses related to opening up. IPR is based on video recordings of sessions to aid clients’ recollection. Important sequences of the session can be played several times to help clients to access, explore, and verbalize visible and covert experiences (Kagan, 1980). In our design, additional data was collected in standard follow-up interviews without video recordings. Each client, from now on termed participant, was invited to participate in two IPR interviews (after session 3/4 and 7/8) and one follow-up interview, three interviews in total, see Figure 1 for an illustration of the design. All interviews were audiotaped and transcribed verbatim for analysis. We expected each of the three serial interviews to contain information about opening up in the initial phase of psychotherapy: IPR1 being collected during the initial phase of therapy, IPR2 presenting residuals of experiences from the early phase of therapy, and the follow-up interview allowing for saturation of former presented information and new reflections. In line with research on alliance formation and conventions in studies of different phases of psychotherapy (Auszra et al., 2013; Malin and Pos, 2015) sessions 1–5 were defined as the initial phase. In our sample, Session 1, and often parts of Session 2, consisted of an intake interview and diagnostic evaluation, because clinics needed to evaluate patients for eligibility. Hence, we chose to target session three as the best approximation to the initial phase of therapy. In a few cases, technical problems or the therapist’s failure to inform the interviewer about the time for a session led to sessions four and eight being used instead of sessions three and seven.

The interviews were conducted primarily by the first author, and the second and third authors conducted two interviews each. The interviews were completed between February 2018 and March 2019.

### Procedure

The IPR interviews and the follow-up interview proceeded as follows. Immediately (90 min) after each therapy session, the participant met with the researcher to watch the video of that session in a designated interview room. During the 90-min pause the interviewer prepared by watching through the session and noted the time of sequences that seemed relevant. In the IPR-interview, the participant was asked to pause the video when he or she recognized that “in some way that a feeling or emotion was present, or could have been present” (see interview guide, Table 1). Each time the participant paused the video, the researcher conducted an in-depth interview of the participant’s internal experiences. The part of the video related to the participant’s experience was replayed several times until the participant stated that he or she did not have more information to add. The interview guide (Table 1) was designed with open-ended questions to help the participant focus on experiences happening at a particular point in therapy, not on explanations or how he or she conceived their experiences in retrospect. Questions were modified and added to help the participant give phenomenologically rich descriptions. The IPR interviews lasted between 50 and 120 min, depending on participant’s tiredness and need for a break. Because of the time restriction of 120 min, no participant reviewed an entire session during an IPR interview.

**TABLE 1:** Interview schedule.

**Introduction Presented to Each Participant Before the IPR Interviews**
Standard introduction to ensure safety and confidentiality. I am interested in moments in which you somehow experience something personal or emotional, or for some reason, you could have experienced something personal or emotional but did not. For example, you might be having an emotional experience, but 1. Felt you did not have time to address this 2. You experienced something that was difficult to verbalize 3. You might have thought that it was not important 4. You might not have wanted to speak about it there and then
**IPR Interview**
After each pause in the video: What did you experience in this moment while sitting in the chair?/How was it for you to sit in the chair now? Was there something else happening inside you? How did the therapist contribute to this? Do you have any thoughts how you contributed to this? Do you view this as beneficial/unbeneficial for treatment?
**Follow-Up Interview**
1. The interviewer presented topics from former interviews, by reading transcribed material from the interview or replaying audio-tapes of the interview. Do you recognize this? How do you view it now? If changed—how do you understand these changes? 2. Do you feel treatment has changed the way you think about or relate to your emotions? How?

The follow-up interview took place 3 months after the last IPR interview, and was not based on video. The main purpose of the follow-up interview was to give participants the opportunity to elaborate upon statements from IPR1 and IPR2, and for the researchers to check back with participants about their preliminary understandings of former accounts. At the same time, participants assessed whether they recognized and agreed with their previous statements or if their experiences had changed. In the follow-up interviews, sections from the IPR interviews in which the clients seemed especially engaged or that represented topics they spent much time on were reread or replayed in audio. The follow-up interviews ranged from 60 to 120 min.

### Data Material

The data material from the 11 participants consisted of 28 transcribed interviews, namely, 20 IPR interviews and eight follow-up interviews (see Figure 1). Parts of the video-recorded session were transcribed and kept as data material, in segments of 2 min before and after the participant stopped the video. This was done to capture the context of the participants’ experiences, in case this was needed to understand participants’ statements in the interviews. This material was not subject for analysis. Seven participants completed three interviews as prescribed. Two participants had symptom elevation during the course of treatment and were referred for hospitalization. In collaboration with the therapist who communicated with these participants, we decided to terminate further data collection for the study, as their therapies was put on hold. These participants had completed three IPR interviews in total. One participant ended treatment after the first IPR interview but completed the follow-up interview. One participant did not show up for the follow-up interview.

### Data Analysis

Analysis was conducted across participants’ accounts to look for commonalities and divergences in their experiences of the process of opening up in the initial phase of therapy. The target phenomena was expected to be addressed in IPR1, IPR2, and the follow-up interview; hence all three interviews were subject for analysis. The data were analyzed through a systematic team-based approach according to the principles of thematic analysis (Braun and Clarke, 2006), establishing themes across the participants’ accounts. The analysis proceeded as follows: (1) all the authors read the material to get a basic sense of its meaning and made preliminary process notes; (2) [GK], [AH], and [CM] met for a two-day analytic seminar to reflect on the data, look for patterns of emerging themes, and organize the focus of further analysis. (3) [GK] used the suggested themes from the seminar and conducted a structured thematic analysis of the data material, using QSR International’s NVivo software, which resulted in a list of nine tentative themes encompassing, in total, 320 nodes of participants’ accounts; (4) [GK] and [CM] met to work through and review the code structure for tentative themes, and further condensation of the material; (5) [GK] refined the analysis and rearranged the themes, and checked back with [CM] and [AH] for correspondence; (6) the final thematic structure alongside the full data material was presented to [MR], who critically audited the analytic process, and (7) the GK conducted a cross-analysis of the final themes by going through all of the coded participants accounts to establish the frequencies of findings within the themes.

### Ethics

This study invited participants to share deeply sensitive information while in a vulnerable situation, and the interview procedure demanded ethical reflection. All the participants signed an informed consent form stating that video and audio recordings would be used. Confidentiality was assured by storing the data material and information about the participants separately. All references to the therapists and participants in the transcripts were anonymized. All participants were informed that the content of the interview would not be discussed with the therapists. Participants were informed that at any point until the publication of scientific papers they could withdraw from the study without consequence. Participants’ safety was ensured by using debriefing strategies after the interviews and the fact that the participants were in active treatment. None of the participants terminated interviews or requested extra follow-up after participating in the interviews. The study was approved by the Regional Ethical Committee in Norway 2017/55 and by the health trust’s data security agent.

### Reflexivity

In this study the interviewer was an active part of the conversation in the interviews. This participation demands high degree of reflexivity, since the interviewer could direct the participant and bias the data. The aim was to explore clients processes across therapeutic orientations, yet, all the main researchers have a disciplinary affiliation to emotion-focused therapy (EFT). Concretely, to avoid EFT terminology and theoretical assumptions affecting clients accounts, the interview guide was designed with an explicit focus on avoiding the use of such. Furthermore, we took precautions to stay as close as possible to the participants’ descriptions of moment-to-moment changes, using their words and making explicit if we added something to their descriptions. The participants’ accounts were continually summarized and checked with the participants throughout and at the end of each IPR interview, and during the follow-up interview. At the follow-up interview, contradictions and ambiguities in the participants’ accounts were also discussed. Throughout the analyses, we actively refrained from using an EFT framework when structuring the material. The analytic process was critically audited by an external researcher, with no affiliation to EFT, to check if findings corresponded with participants’ accounts.

### Report Conventions

To report the frequency of specific findings across the participants accounts, we use the recommendations of Hill et al. (2005). *General* refers to all participants or all participants but one (10 ≤ *n* ≤ 11), *typical* includes more than half of the participants (6 ≤ *n* ≤ 9), while *variant* includes at least two of the participants up to half of the participants (2 ≤ *n* ≤ 5).

## Results

### Main Theme: Holding Back and Struggling to Open Up

Participants shared information about the process of opening up in the initial phase of therapy in all three of the serial interviews. Generally, participants spoke about hesitation, worry, and insecurity when they recognized that they had the possibility to open up, both in IPR1 and IPR2. The main theme was labeled “Holding back and struggling to open up.” Four subthemes constituted the theme of holding, or feeling held back;

1. Fearing the intensity and consequences of negative emotions.
2. Experiences of being incapable and bodily stuck.
3. Being insecure about one’s worthiness and right to share inner experiences with the therapist.
4. Struggling with feeling disloyal to loved ones

When participants reviewed the sessions on screen in IPR1 and IPR2, they generally discovered that they let out emotions only partially, if at all. The participants generally discovered moments in which they distanced themselves from inner experiences, either by the means of obvious strategies such as telling anecdotes of lesser personal and self-deprecating character, avoiding eye-contact, or less overt processes such thinking about everyday matters and inner discussion on whether to open up or not. The participants differed greatly in how they experienced struggles over opening up at the initial phase of therapy; this will be presented in the following four subthemes.

#### Fearing the Intensity and Consequences of Negative Emotions

The first theme pertains to participants’ experiences of holding back because of fear and worry about what could happen if they opened up and shared important feelings with the therapist. A variant number of participants first became aware of their hesitations or reluctance while watching the video. Avoiding emotions was typically described as intentional, hard work or variantly as a habitual tendency. One participant stopped the video in a sequence where she sat with her eyes closed when the therapist explicitly focused on becoming aware of inner experiences, using a focusing intervention. The participant said in the interview that she was more or less pretending to do the exercise in the session. In the IPR interview she explored why she worked against the purpose of the intervention:

Feelings are scary, and, and, uhm, especially those that aren’t. those that you don’t understand, too. It’s as if he’s asking me to jump into a black hole. It’s like you don’t know what’s coming. what’s on the other side. (.) the biggest and scariest unknown (Participant 09, IPR1).

Participants’ anticipations consisted primarily of worries or fear of psychological pain; for example, becoming overwhelmed, helpless, chaotic, embarrassed, and ashamed, both in session and afterward. Fear of losing control was a recurrent theme. This fear was described variantly as strong, resembling symptoms of panic attack. The participants used a variety of metaphors to describe their anticipation of opening up to emotions; for example, comparing it to the act of opening a vent or having a bomb explode. In the interview, participants generally made a point of having stored and carried a load of painful experiences over time, experiences that had never been properly let out and worked through. Consequently, this led to a fear of the intensity of these emotions. Participants also typically explained that it was too difficult to bring certain topics to life; they were not ready to face their challenging past or the feelings of sadness or shame that burdened them. Generally, the participants reported experiencing that sharing emotions had led to criticism, shame, and increased loneliness.

One participant explored a video sequence where the therapist commented on a story about how her parents had not stood up for her as a child. In one session, after trying to explain the situation in further detail, she was encouraged to participate in a focusing exercise, by giving attention to bodily experiences, and not putting words to it. In the interview, she described that during the exercise she tried to stop the tears that started to appear. In the interview, she envisioned the consequences of not holding back:

I imagine these sorts of rivers of something yucky just being spewed out. Err, venom, bile, and evil. Everything that I haven’t been able to get out. And that it all comes at once and almost chokes both me and my surroundings (.). So I, I think it might be that there’s a lot simmering beneath the surface in me. Much more than I’m aware of. And that frightens me (Participant 7, IPR1).

These accounts showed the participants’ tendency to view themselves as passive receivers of their negative emotions.

Participants variantly shared worries about becoming a negative version of themselves if they opened up. For example, being sincere about anger could potentially reveal a predisposition to physical violence. Some participants shared worries about becoming manic or depressed, having a mental breakdown, or perhaps being in need of hospitalization. Participants variantly reported worries about becoming stuck in their emotions for eternity:

If you accept your feelings, then the feelings will take over, and if you accept the anger, or the grief, or whatever it may be, then you will somehow drown in it and never get out again. (.) I had that fear.—I was completely convinced that that’s how it would be (Participant 10, FU1).

These statements revealed insecurity connected to the project of psychotherapy. In initial phases, participants variantly worried that going into troublesome topics would not be helpful, and that it even could make them feel worse.

#### Experiences of Being Incapable and Bodily Stuck

The second theme covers clients’ difficulties in opening up because of experiences of insecurity, incompetence, lack of words to verbalize inner processes, and experiences of the body halting the process. In the initial phase of therapy, when the therapists asked questions along the lines of “How does that make you feel?” participants would struggle to understand what the therapists were aiming at. Variantly, participants said that they became stressed or felt inadequate because they believed they did not deliver what their therapist required. Participants also variantly revealed that they became annoyed by the therapist repeatedly asking about inner experiences when they had stated several times that they did not know. One participant was aware that the therapist tried to make her get in touch with her feelings because the therapist repeatedly asked questions such as “Where in the body do you feel it?” She did get in touch with a new and alien feeling in session, but she answered the therapist that she did not know. She explained in the interview that she needed to be confident and sure about the feeling before she could share it with the therapist.

A general statement was the lack of former experience addressing inner experiences in life as a whole. This contributed to feeling incapable as a client.

The participants gave many different bodily descriptions of experiences or feelings of being stuck; for example, “words being stuck in the throat” (Participant 10, IPR1), “feelings being swallowed” (Participant 7, IPR1) and *“*the body becoming locked” (Participant 5, IPR1). Typically the participants would point to the chest or mouth during the interview, explaining that during the session they wanted to pull something out. A participant said to her therapist in the beginning of the fourth session that she had been thinking that she maybe did not need to continue treatment. The therapist asked: “So you have been feeling very well, then?” The participant stopped the video in the middle of her answer about things going better, and shared her inner experience:

Yes, here! Here. here I’m, I’m struggling with wanting to say that my mother has gone to the hospital, but then there’s something inside me stopping me and trying to. it keeps holding back, and then I don’t understand why I’m holding it back. (.) But, it was a very odd feeling, it was as if I was thinking. you can hear that I’m hesitating, and that’s exactly because it’s as if I open my mouth to say it, but, but, then. the words just go silent inside me. And I’m sort of, yeah, really sort of held back, almost like one of those harn. yeah, harnesses that pulls back. inside me, and then I don’t understand why it’s happening either, and then I get a bit stressed and frustrated by it, too (Participant 10, IPR1).

By these accounts, the participants stated experiences and feelings as embodied, and that feelings or experiences appeared like *an object* with the potential of being released from the body.

Several participants reported that it seemed as if their mind and body did not cooperate when they encountered highly sensitive issues in sessions. A woman had as an explicit goal to let down her mask, and she commented while exploring a challenging part of a session: “I’m sitting there doing something my entire body is fighting against and wants to escape from. I mean, your body wants to get away, but I want to work with it. So, there’s a huge inner conflict” (Participant 08, IPR1). Participants accounts included in this subtheme typically reported that arising feelings suddenly or gradually vanished. Participants variantly spoke about their factual knowledge and feelings not corresponding, illustrated by the following quote:

I know that that therapist doesn’t mean there is no hope me, but my body interprets his words differently (Participant 2, IPR1).

#### Being Insecure About One’s Worthiness and Right to Share Inner Experiences With the Therapist

Variantly participants were not sure if their personal issues were relevant and important enough to bring up in therapy. They could question if they exaggerated or perceived their experiences in a faulty way. Participants noticed sequences in the sessions in which they were downplaying the importance of their own experiences or point of view. In this context, downplaying was done by making their own experiences less important than those of other persons involved, blaming themselves for interpersonal problems or finding excuses for their feelings. One participant stopped the video and said that she felt discomfort in the session because the therapist looked sad when she talked about the difficulty of making her family understand her, and she felt the need to protect the therapist:

Participant: I try not to make it sound so. so sad, serious, or so bad. (.) Uhm, because. to me it looks like she gets upset, somehow, yeah. It. I don’t want that, of course. I don’t want other people, how to put this, to suffer. (.) Yeah, I couldn’t physically remove myself from the situation, so (…) I have to. think about what to say, so it’s a bit like. stupid and in a way, because I then kind of have to control my feelings (Participant 5, IPR1).

Variantly, participants explained their tendency not to take their own experiences into account as a consequence of not being heard earlier in life—they had learned that their feelings were not that important. This tendency was typically described as automatic. One participant thought she held back when she could have shown emotions because this was a social norm: *You are supposed to just pull yourself together* (Participant 9, IPR1).

Participants typically recognized that they felt embarrassed and ashamed over still struggling with certain life events or emotions. In the interview, they stated that they should have been able to let go a long time ago or been able to manage their difficulties on their own. One elderly participant teared up in the interview when she watched herself mentioning a difficult childhood experience. In the session, she was vaguely smiling. She said that in the session she felt the incident was something that she at her age should not be affected by:

Participant: I feel that (…) these are things that really should’ve been forgotten a long time ago. And perhaps it had been forgotten for many years. But it’s. the feeling that I’ve never been good enough has stayed with me.

Interviewer: What is it like for you? Does it do something to you?

Participant: It’s actually a bit painful to watch.

Interviewer: But, in that video, can you remember whether it did something to you there and then?

Participant: Not really, but now I’m thinking that this really wasn’t OK. (Participant 6, IPR2).

In the first round of IPR interviews, participants variantly pointed to a common phenomenon, that the therapist relationship did not feel safe enough for them to open up about personal and sensitive topics. Participants also variantly underscored that they felt safe to open up—to “let down their guard” (Participant 10, IPR1), to “let the therapist see behind the mask” (Participant 9, IPR1), or to “just be oneself” (Participant 8, IPR1). These latter participants believed their struggles to open up were not related to feeling unsafe or uncomfortable *per se* in front of their therapist. As one participant explained:

Even if it’s not dangerous to talk to the therapist about your feelings, your being remembers that it’s been difficult so many times that you automatically stop yourself. (.) i.e., that you’ve learned to live and be that person. I’m not worried that [name of therapist] will let me down, but my entire body tells me that “whoa, suddenly there’s an opening in the conversation, if I go down this path, I cannot get out of it.” (Participant 9, IPR2).

#### Struggling With Feeling Disloyal to Loved Ones

The final theme described the difficulty the participants experienced when they brought up issues that involved persons who were close to them. In IPR1, participants generally conveyed feeling guilty and ashamed when they mentioned family members or important others. Participants generally stated that it intuitively felt wrong to open up about and be specific about troubles related to family. These feelings of disloyalty were rarely presented to the therapist in the ongoing sequences. One person stated in the follow-up interview that the storm of emotions she had felt at the beginning of therapy probably was due to feeling tremendously disloyal to her family. She explained, further on, that several times she regretted starting therapy because she felt uncomfortable sharing something that could be perceived as criticism of her family members. The participants experienced speaking about significant others as challenging, and this could hinder the process of sharing sensitive personal information or exploring their own feelings. Participants typically discovered when watching the video that they automatically protected others rather than focusing on their own inner experiences—experiences like being hurt, unfairly treated, or upset. One participant worked with her difficult relationship with her mother in therapy, but this would also lead to talking about other family members. In the first IPR interview, she quickly recognized feelings of discomfort as soon as she mentioned the names of loved ones in the session:

I feel bad about talking about people who aren’t in the room. I find it difficult. And that can absolutely be a barrier, right, to. how I proceed from there. If I. can’t. because then there might be situations that I wouldn’t have wanted to raise with him.

Interviewer: What could it be a barrier to?

Participant: Maybe just saying things how they are. Yeah, I’m more concerned about protecting the people I’m talking about, or those who were present, than about actually saying how I feel (Participant 4, IPR1).

Instead of focusing on their own experiences when this would have been natural, participants noticed themselves spending time explaining the surrounding family context in great detail. They explained that they needed to make sure that the therapist did not get the wrong picture of those close to them; it was important not to do an injustice to others. Some participants stated that protecting others did not necessarily keep them from sharing more about their own inner experiences in the long run, but it was important that they felt safe that the therapist knew “the whole picture” in preparation to their more fully opening up.

The participants typically revealed having mixed feelings toward people who were or had been a big part of their life. Feelings of disloyalty became apparent even among participants who stated they had clear reasons for having negative feelings toward others (for example, having been subjected to severe forms of maltreatment by caregivers), and among participants who were not fully convinced their feelings were justified. Many participants described the process of admitting negative feelings toward caregivers as tremendously challenging. One participant, who was on the verge of starting to share very personal information from childhood for the first time, explained:

Imagine if he [close associate] were to hear some of this? Do I dare say it out loud? What if someone hears me? I’m actually thinking that during the conversation. Should I talk about this? Can I talk about this? Is it, you know, normal? But then I think I’m in a safe setting. But what will he [name of therapist] think about me if I say it? I thought about that for a moment. But then I think, “No, it’s not like that.” But those thoughts are there (Participant 3, IPR2).

## Discussion

The findings provide insight into clients’ internal experiences when holding back or feeling held back, on a moment-to-moment basis in the initial stages of therapy. Across subthemes, participants reported tension, because they generally acknowledged the potential gain of opening up, but instead ended up distancing themselves from or not sharing inner experiences. These tensions typically made starting therapy a challenging experience. Participants experienced the need to be a good client and to benefit from therapy, but were concerned about what conduct was appropriate in a therapeutic setting: did they display too little emotion or did they display too much? Were they expected to control their feelings (themes one and two)? Which experiences were relevant for therapy (theme three) and how can one find a way of working with personal issues, while at the same time not being too critical or disloyal to loved ones (theme four). These results underscore how coming to a therapy process is connected to apprehension and a need to discover what the social rules of the situation are, a point recently emphasized in a qualitative meta-analysis of clients’ experiences with starting therapy from an alliance formation perspective (Lavik et al., 2018).

From this start, both intrapsychic processes and interpersonal processes contributed to holding back across the reported themes. Some participants were concerned with the experience of sharing personal matter in front of the therapist, others had personal blocks that hindered them from approaching emotions, irrespective of the therapist’s presence. Several references to shame were embedded within all of the four subthemes, indicating the importance of the relational tension in session. Indeed, shame is often related to feeling inferior and socially unattractive, and it tends to coincide with an urge to conceal, hide, and cover up (Gilbert, 2011), hence, making the initial phases of therapy potentially difficult.

Our findings correspond with the research of Rennie (1994a), Levitt (2001), and Frankel and Levitt (2009) which shows that clients’ internal struggles when exploring experiences, or attempting to share experiences with the therapist, can result in defensive activities such as disengagement (Holdsworth et al., 2014) and deference (Rennie, 1994b). Client involvement and avoidance-related factors in therapy have been recognized as common across theoretical orientations (Bohart and Tallman, 2010). Yet empirical research on resistance interventions and the effect of avoidance on therapeutic progress is scarce (Holtforth, 2008). The present study contributes by studying these processes *in vivo*, and thereby having a potential contribution by contextualizing them.

Our study contextualizes concepts of resistance and avoidance by exploring how and why clients withhold emotional difficulties due to insecurities or fears connected to what is appropriate in therapy. The subthemes describe important concerns clients might hold. Our results suggest that it important for therapists to be attentive to these concerns and to validate to ambiguous feelings tied to entering a client role. Without this awareness, we risk that clients internalize a situational difficulty as a personal flaw and reduce their hope that therapy might help them. As such, holding back can be conceived of as a natural phenomenon in initial stages of therapy that could be addressed, validated and worked through. For some clients, a process of holding back can be a necessary first step of working through relevant issues.

### Is Holding Back an Automatic Response or a Deliberate Choice?

An important question emerging in the analyses was whether holding back was experienced by participants as a deliberate, intentional choice or as an automatic, unintentional act. The findings showed that participants do not always have accurate knowledge of how hesitant they are when encountering sensitive material in the initial phase of psychotherapy. During the video-assisted in-depth interviews, participants reported visible and internal ways of holding back to be much more extensive than they had imagined before discovering it on the screen. Multiple vivid revisits of incidents in sessions made participants realize how skilled or accustomed they were at distancing themselves from challenging topics and avoiding difficult emotions. From a data analysis point of view, it is difficult to conclude whether the experience of holding back was already present in the participant during the session, or if that is the interpretation that participants develop in the IPR-interview when they see the discrepancy between how they knew they felt and what they expressed on the outside in the video. However, in the process of meaning making shortly after the session, understandings such as “this happens automatically” and “here I actively choose to hold back” seemed to represent the participants’ experiences and vary between situations.

The theme “Being incapable and bodily stuck” described a distinct experience of physical arrest. When emotional reactions started to erupt, the participants felt that their body held them back. This was perceived by many as an automatic event that occurred both in situations in which participants privately dealt with difficult topics, and when they shared their experiences with the therapist. Several participants described tensions between wanting to open up and the body automatically pulling away. Importantly, these finding illustrate how defense mechanisms in psychotherapy can be perceived by participants as solely embodied phenomena that are difficult to change.

Further on, participants were often aware that the therapist tried to help them engage with inner experiences, but thought it was themselves who halted the process. It simply seemed unavoidable to distance themselves from inner experiences, no matter how the therapist dealt with these moments. The tendency to hold back was viewed by many as habitual, and potentially connected to former life experiences. This experience of holding back as being unavoidable is in line with findings from an IPR study by Frankel and Levitt (2009) on clients’ experiences of moments of disengagement in psychotherapy. Participants in this study described disengagement as an automatic, unintentional reflex that was initiated when they needed to protect themselves from potential discomfort and feelings of loss of control.

On other occasions, the participants also reported holding back as intentional and hard work, both psychologically and physically, especially in the theme “fearing the intensity and consequences of negative emotions”. This theme included illustrations of participants making more deliberate choices of whether to hold back or not, as they reflected on their readiness to move into more challenging topics. This seems to underscore the understanding that holding back can be motivated both by prosocial interpersonal sensitivity and unfamiliarity with the client role, as well as intrapsychic difficulty in its own right.

Holding back as both intentional and as something that the participant could not control, corresponds with Levitt (2001) results in a study on “obstructive pauses” in psychotherapy. In Levitt’s study, participants reported avoiding painful emotions in different ways: they could distance themselves by actively avoiding entering a deep feeling, using apparent strategies as for example distracting the therapist. Or, they could more passively end up feeling distant and withdrawn, by “shutting down,” thinking that they had lesser control over the process.

### How Are Participants’ Experiences of Holding Back in the Initial Phase of Psychotherapy Important?

The findings shed light on processes that might be involved when clients experience dissatisfaction in psychotherapy, and when they don’t think they benefit from treatment. In worst case scenario these clients drop out of psychotherapy, which is an established concern (Swift and Greenberg, 2012). Moreover, unique experiences of holding back are important areas to focus on in the initial phase of therapy, when the therapist tries to establish the safety required for the client to enter challenging emotional experiences. If the internal struggles accounted for in this study are sufficiently addressed, in accordance with emotion processing theories, later mastering of negative feelings can become easier. Therapists should be aware of the range of concerns experienced by clients that lead to holding back. Moreover, research has demonstrated that patients withdrawing or disengaging from the therapeutic encounter can be frustrating for therapists (Moltu et al., 2010; Ribeiro et al., 2014). Therapists who are caught in frustrating counter-transferential reactions risk being less helpful, acting out frustrated feelings or withdrawing their open presence to the clients (Hayes et al., 2011). Client defensiveness can sometimes colloquially be talked about as a client being difficult or hard to treat, a form of talk that might indicate that the empathic attunement to the client’s perspective is threatened, sometimes referred to as “blaming the client” for the lack of therapeutic success. Natvik and Moltu (2016) discussed how phenomenologically based research could be helpful in this regard, by allowing for a lived engagement with the client’s perspective. The client perspective can support the clinical reader in meeting with conceptual knowledge, for example theoretical concepts that bear resemblance to processes of holding back (e.g., “experiential avoidance” Hayes and Wilson, 1994).

### Implications

Ideally, therapists should take clients’ immediate experiences when holding back into account when setting the stage for psychotherapeutic work. However, as the findings of this study show, clients might not be ready or inclined to share inner experiences during the initial phase of therapy. The act of holding back is often unspoken and far from obvious, and in this vein, this study contributes valuable clinical knowledge about clients’ inner experiences when holding back. One core implication from this study is the suggestion that therapist training in flexible attunement and methods for inquiry into clients’ inner experiences in the initial phase of therapy are important across therapeutic modalities. First, knowledge that allows therapists to empathize with the client’s reasons for disengaging, such as uncertainty about client role expectations and loyalty conflicts to loved ones, might support the alliance and validation processes in early phases of therapy. Therapist activities that address these processes successfully could be a fruitful avenue for future research. Second, communication that helps clients becoming aware of habitual and embodied ways of shutting down experience seems one important part of a general therapeutic skillset.

### Limitations and Strengths

The clinical sample in this study was small, and none of the therapists stated they used non-experiential treatment approaches such as cognitive behavioral therapy. Inclusion of participants in non-experiential treatment could potentially contribute accounts adding to the established themes or result in other added themes. Participant bias in form of giving information in line with what is believed to be the researchers’ or therapists’ expectation might have occurred. The recruiters may have been influenced by the information they received about the study involving clients experiences of emotion in therapy, to change the focus of the sessions or recruit participants they assumed would be suited to the project, contrary to instructions. From a procedures perspective, the IPR interviews did not allow for examinations of the full therapeutic session. Consequently, we have studied in depth more experiences from the beginning half than from the last half of the session. In terms of opening up, this might have led to relevant experiences in the end of sessions being missed. To counter this potential limitation, the interviewer reviewed the full session in preparation for the interview, in case significant shifts that needed attention were present late in the session. Based on this safeguard we do not expect that some experiences are systematically left out from the data. Moreover, combining interviews based on subjective explorations with structured observer ratings of sessions could add an extra perspective. Based on these limitations, one should be careful in generalizing from these results alone.

The IPR method is based on participants report, and this makes response-bias an relevant issue. Moreover, the IPR inquiry relies on the participant’s capacity to recognize and report subjective perspectives, and that they feel comfortable sharing personal information. Yet the second IPR interview and the follow-up interview increased the potential for reaching fuller and more nuanced descriptions of the participants’ experiences because the participants were more familiar with the focus of the research questions and were more accustomed to the therapy situation.

Using a microanalytic process design to retrieve in-depth descriptions of aspects related to the processes in holding back has great promise, not only by stimulating recall, but by increasing participants’ ability to acquire a detached frame of reference when describing internal processes. Further microprocess research is needed to increase our understanding of the experiences accompanying the clients’ tendency to hold back. This can, in turn, inform relevant interventions that can help clients to move from holding back to opening up in pivotal moments in the initial phase of psychotherapy.

## Data Availability Statement

The anonymized raw data supporting the conclusions of this article will be made available by the authors, without undue reservation, to any qualified researcher within the boundaries of data storage approvals.

## Ethics Statement

The studies involving human participants were reviewed and approved by the Regional committees for medical and health research ethics Norway (REC West). The patients/participants provided their written informed consent to participate in this study. Written informed consent was obtained from the individual(s) for the publication of any potentially identifiable images or data included in this article.

## Author Contributions

GK was the project leader and main author of the article. GK, CM, and AH conducted the interviews and analyzed the data material. MR critically audited the analytic process. All authors contributed to the article and approved the submitted version.

## Conflict of Interest

The authors declare that the research was conducted in the absence of any commercial or financial relationships that could be construed as a potential conflict of interest.

## References

[B1] American Psychological Association, Presidential Task Force on Evidence-Based Practice (2006). Evidence-based practice in psychology. *Am. Psychol.* 61 271–285. 10.1037/0003-066X.61.4.271 16719673

[B2] Auszra L. Greenberg L. S. Herrmann I. (2013). Client emotional productivity-optimal client in-session emotional processing in experiential therapy. *Psychother. Res.* 23 732–746. 10.1080/10503307.2013.816882 23848974

[B3] Barbosa E. Amendoeira M. Bento T. Teixeira A. Pinto-Gouveia J. Salgado J. (2017). Immersion and distancing across the therapeutic process: relationship to symptoms and emotional arousal. *Res. Psychother. Psychopathol. Process Outcome* 20 110–121. 10.4081/ripppo.2017.258 32913739 PMC7451309

[B4] Baumann E. C. Hill C. E. (2016). Client concealment and disclosure of secrets in outpatient psychotherapy. *Counsel. Psychol. Q.* 29 53–75. 10.1080/09515070.2015.1023698

[B5] Bohart A. C. Tallman K. (2010). “Clients: the neglected common factor in psychotherapy,” in *The Heart and Soul of Change: Delivering What Works in Therapy*, 2nd Edn, eds Duncan B. L. Miller S. D. Wampold B. E. Hubble M. A. (Washington, DC: American Psychological Association), 83–111. 10.1037/12075-003

[B6] Bohart A. C. Wade A. G. (2013). “The client in psychotherapy,” in *Bergin and Garfield’s Handbook of Psychotherapy and Behavior Change*, 6th Edn, ed. Lambert M. J. (Hoboken, NJ: John Wiley & Sons, Inc), 219–257.

[B7] Braun V. Clarke V. (2006). Using thematic analysis in psychology. *Qual. Res. Psychol.* 3 77–101. 10.1191/1478088706qp063oa 32100154

[B8] Diener M. J. Hilsenroth M. J. Weinberger J. (2007). Therapist affect focus and patient outcomes in psychodynamic psychotherapy: a meta-analysis. *Am. J. Psychiatry* 164 936–941. 10.1176/ajp.2007.164.6.936 17541054

[B9] Elliott R. (1986). “Interpersonal process recall (IPR) as a psychotherapy process research method,” in *The Psychotherapeutic Process: A Research Handbook*, eds Greenberg L. S. Pinsof W. M. (New York, NY: Guilford Press), 503–527.

[B10] Farber B. A. (2003). Patient self-disclosure: a review of the research. *J. Clin. Psychol.* 59 589–600. 10.1002/jclp.10161 12696134

[B11] Flückiger C. Del Re A. C. Wampold B. E. Horvath A. O. (2018). The alliance in adult psychotherapy: a meta-analytic synthesis. *Psychotherapy* 55 316–340. 10.1037/pst0000172 29792475

[B12] Frankel Z. Levitt H. M. (2009). Clients’ experiences of disengaged moments in psychotherapy: a grounded theory analysis. *J. Contemp. Psychother.* 39 171–186. 10.1007/s10879-008-9087-z

[B13] Gelso C. J. Kivlighan D. M. Jr. Markin R. D. (2018). The real relationship and its role in psychotherapy outcome: a meta-analysis. *Psychotherapy* 55 434–444. 10.1037/pst0000183 30335456

[B14] Gilbert P. (2011). “Shame in psychotherapy and the role of compassion focused therapy,” in *Shame in the Therapy Hour*, eds Dearing R. L. Tangney J. P. (Washington, DC: American Psychological Association), 325–354. 10.1037/12326-014

[B15] Greenberg L. S. Goldman R. N. (2019). “Theory of practice of emotion-focused therapy,” in *Clinical Handbook of Emotion-Focused Therapy*, eds Greenberg L. S. Goldman R. N. (Washington, DC: American Psychological Association), 61–89. 10.1037/0000112-003

[B16] Hayes J. A. Gelso C. J. Hummel A. M. (2011). “Managing countertransference,” in *Psychotherapy Relationships that Work: Evidence-Based Responsiveness*, 2nd Edn, ed. John N. C. (New York, NY: Oxford University Press), 239–258. 10.1093/acprof:oso/9780199737208.003.0012

[B17] Hayes S. C. Wilson K. G. (1994). Acceptance and commitment therapy: altering the verbal support for experiential avoidance. *Behav. Analyst.* 17 289–303. 10.1007/BF03392677 22478193 PMC2733476

[B18] Hill C. E. Knox S. Thompson B. J. Williams E. N. Hess S. A. Ladany N. (2005). Consensual qualitative research: an update. *J. Counsel. Psychol.* 52 196–205. 10.1037/0022-0167.52.2.196

[B19] Holdsworth E. Bowen E. Brown S. Howat D. (2014). Client engagement in psychotherapeutic treatment and associations with client characteristics, therapist characteristics, and treatment factors. *Clin. Psychol. Rev.* 34 428–450. 10.1016/j.cpr.2014.06.004 25000204

[B20] Holtforth M. G. (2008). Avoidance motivation in psychological problems and psychotherapy. *Psychother. Res.* 18 147–159. 10.1080/10503300701765849 18815972

[B21] Kagan N. (1980). “Influencing human interaction eighteen years with IPR,” in *Psychotherapysupervision: Theory, Research, and Practice*, ed. Hess A. K. (New York, NY: Wiley), 262–283.

[B22] Kolden G. G. Wang C.-C. Austin S. B. Chang Y. Klein M. H. (2018). Congruence/genuineness: a meta-analysis. *Psychotherapy* 55 424–433. 10.1037/pst0000162 30335455

[B23] Lane R. D. Ryan L. Nadel L. Greenberg L. (2014). Memory reconsolidation, emotional arousal, and the process of change in psychotherapy: new insights from brain science. *Behav. Brain Sci.* 38:e1. 10.1017/S0140525X14000041 24827452

[B24] Lavik K. O. Frøysa H. Brattebø K. F. McLeod J. Moltu C. (2018). The first sessions of psychotherapy: a qualitative meta-analysis of alliance formation processes. *J. Psychother. Integrat.* 28:348 10.1037/int0000101

[B25] Levitt H. M. (2001). Clients’ experiences of obstructive silence: integrating conscious reports and analytic theories. *J. Contemp. Psychother.* 31 221–244. 10.1023/A:1015307311143

[B26] Levitt H. M. (2002). The unsaid in the psychotherapy narrative: voicing the unvoiced. *Counsel. Psychol. Q.* 15 333–350. 10.1080/0951507021000029667

[B27] Levitt H. M. Pomerville A. Surace F. I. (2016). A qualitative meta-analysis examining clients’ experiences of psychotherapy: a new agenda. *Psychol. Bull.* 142 801–830. 10.1037/bul0000057 27123862

[B28] MacFarlane P. Anderson T. McClintock A. S. (2015). The early formation of the working alliance from the client’s perspective: a qualitative study. *Psychotherapy* 52 363–372. 10.1037/a0038733 25706060

[B29] Malin A. J. Pos A. E. (2015). The impact of early empathy on alliance building, emotional processing, and outcome during experiential treatment of depression. *Psychother. Res.* 25 445–459. 10.1080/10503307.2014.901572 24801633

[B30] Marks E. C. Hill C. E. Kivlighan D. M. Jr. (2019). Secrets in psychotherapy: for better or worse? *J. Counsel. Psychol.* 66 70–82. 10.1037/cou0000311 30299124

[B31] McAleavey A. Castonguay L. G. (2015). “The process of change in psychotherapy: common and unique factors,” in *Psychotherapy Research*, eds Gelo O. Pritz A. Rieken B. (Cham: Springer). 10.1007/978-3-7091-1382-0_15

[B32] Moltu C. Binder P.-E. Nielsen G. H. (2010). Commitment under pressure: experienced therapists’ inner work during difficult therapeutic impasses. *Psychother. Res.* 20 309–320. 10.1080/10503300903470610 20099206

[B33] Natvik E. Moltu C. (2016). Just experiences? Ethical contributions of phenomenologically-oriented research. *Scand. Psychol.* 3:e17 10.15714/scandpsychol.3.e17

[B34] Pascual-Leone A. Yeryomenko N. (2017). The client “experiencing” scale as a predictor of treatment outcomes: a meta-analysis on psychotherapy process. *Psychother. Res.* 27 653–665. 10.1080/10503307.2016.1152409 26961204

[B35] Peluso P. R. Freund R. R. (2018). Therapist and client emotional expression and psychotherapy outcomes: a meta-analysis. *Psychotherapy* 55 461–472. 10.1037/pst0000165 30335458

[B36] Rennie D. L. (1994a). Clients’ accounts of resistance in counselling: a qualitative analysis. *Can. J. Counsel.* 28 43–57.

[B37] Rennie D. L. (1994b). Clients’ deference in psychotherapy. *J. Counsel. Psychol.* 41 427–437. 10.1037/0022-0167.41.4.427

[B38] Rennie D. L. (1994c). Storytelling in psychotherapy: the client’s subjective experience. *Psychother. Theory Res. Pract. Train.* 31 234–243. 10.1037/h0090224

[B39] Ribeiro A. P. Ribeiro E. Loura J. Gonçalves M. M. Stiles W. B. Horvath A. O. (2014). Therapeutic collaboration and resistance: describing the nature and quality of the therapeutic relationship within ambivalence events using the therapeutic collaboration coding system. *Psychother. Res.* 24 346–359. 10.1080/10503307.2013.856042 24295233

[B40] Rodgers B. Elliott R. (2015). “Qualitative methods in psychotherapy outcome research,” in *Psychotherapy Research: Foundations, Process, and Outcome*, eds Gelo O. C. G. Pritz A. Rieken B. (Cham: Springer), 559–578. 10.1007/978-3-7091-1382-0_27

[B41] Sloan D. M. Kring A. M. (2007). Measuring changes in emotion during psychotherapy: conceptual and methodological issues. *Clin. Psychol. Sci. Pract.* 14 307–322. 10.1111/j.1468-2850.2007.00092.x

[B42] Subic-Wrana C. Greenberg L. S. Lane R. D. Michal M. Wiltink J. Beutel M. E. (2016). Affective change in psychodynamic psychotherapy: theoretical models and clinical approaches to changing emotions. *Zeitschrift Psychosom. Medizin Psychother.* 62 207–223. 10.13109/zptm.2016.62.3.207 27594599

[B43] Swift J. K. Greenberg R. P. (2012). Premature discontinuation in adult psychotherapy: a meta-analysis. *J. Consult. Clin. Psychol.* 80 547–559. 10.1037/a0028226 22506792

[B44] Swift J. K. Tompkins K. A. Parkin S. R. (2017). Understanding the client’s perspective of helpful and hindering events in psychotherapy sessions: a micro-process approach. *J. Clin. Psychol.* 73 1543–1555. 10.1002/jclp.22531 29044600

[B45] Timulak L. Keogh D. (2017). The client’s perspective on (experiences of) psychotherapy: a practice-friendly review. *J. Clin. Psychol.* 73 1556–1567. 10.1002/jclp.22532 28898410

[B46] Whelton W. J. (2004). Emotional processes in psychotherapy: evidence across therapeutic modalities. *Clin. Psychol. Psychother.* 11 58–71. 10.1002/cpp.392

